# New Cure for Gonorrhea

**Published:** 1896-08

**Authors:** 


### New Cure for Gonorrhea.

Under this title the Paris correspondent of the medical press gives an abstract of a lecture by Dr. Routier, of the Hospital Cochin-Routier, says: "Where the gonorrhea is in its acute stage I would advise you not to interfere. Tell the patient to wear a suspensory bandage, to take an alkaline bath every three days, and in about ten days afterward to come back, and then you will cure him. On the other hand, if the case is one that has been of some weeks' standing, the treatment can be commenced at once. Your line of conduct will be guided according to the extent of the affection. If the virus has only attacked the anterior portion of the canal, large irrigations, without forcing the sphincter, will be sufficient, but if the whole of the urethra is affected, urethro-vesical irrigations must be made. In every case accompanied by fistula, abscess, or other complication, the treatment should be directed to that end before attempting to cure the gonorrhea. The modus operandi of the treatment is easy, but essential to follow in detail. A reservoir ad hoc, holding about two quarts, is filled with the solution of permanganate of potash (1 to 2C00), warm, if possible. An India-rubber tube of about two yards in length, and terminated with a glass nozzle, is attached to the reservoir. The patient urinates immediately before the operation so as to wash out any pus that may have collected in the canal since the last micturition. The reservoir being placed about five feet above the penis, the glans and meatus are carefully irrigated, and then the canula is inserted into the meatus, and the liquid flows in until it reaches the sphincter, when it returns and escapes, and so washes out the anterior portion of the urethra. To attain the posterior portion the fingers press the lips of the meatus against the canula, and the liquid not being able to return forces the sphincter and penetrates into the bladder. As soon as the patient feels the want to urinate the current is turned off, and the man recommended to press on the penis from time to time, while rejecting the solution to stop the flow, by which means the liquid penetrates into all the glands, and its action is increased. The operation can be repeated once or twice. If the liquid can not enter the bladder with a pressure of five feet, the reservoir can be placed a little higher, for the resistance of the sphincter varies. If the patient is nervous a catheter can be passed, and the nozzle fixed to it. One seance a day is sufficient. At the end of eight days your patient will be cured, on the condition that he comes every day to be treated. I have thus treated, in the last few weeks, twenty-five cases of gonorrhea, of which eight were in the acute stage, and all of them were cured in a week. In order to ascertain if the patient is really cured of his gonorrhea, instill into his canal a few drops of a solution of nitrate of silver (1 to 1000); a chemical inflammation is provoked, and if any gonococci have remained, a drop of pus will be found in the morning."—*Medical Record.*

